# Long-Term Outcome in Patients with a Solitary Peutz-Jeghers Polyp

**DOI:** 10.1155/2019/8159072

**Published:** 2019-09-08

**Authors:** Masaya Iwamuro, Yuki Aoyama, Seiyuu Suzuki, Sayo Kobayashi, Tatsuya Toyokawa, Yuki Moritou, Shinichiro Hori, Kazuhiro Matsueda, Masao Yoshioka, Takehiro Tanaka, Hiroyuki Okada

**Affiliations:** ^1^Department of Gastroenterology and Hepatology, Okayama University Graduate School of Medicine, Dentistry and Pharmaceutical Sciences, Okayama 700-8558, Japan; ^2^Department of Gastroenterology, Kagawa Prefectural Central Hospital, Takamatsu 760-8557, Japan; ^3^Department of Gastroenterology, Sumitomo Besshi Hospital, Niihama 792-8543, Japan; ^4^Department of Internal Medicine, Fukuyama City Hospital, Fukuyama 721-8511, Japan; ^5^Department of Gastroenterology, Fukuyama Medical Center, Fukuyama 720-8520, Japan; ^6^Department of Internal Medicine, Hiroshima City Hiroshima Citizens Hospital, Hiroshima 730-8518, Japan; ^7^Department of Endoscopy, Shikoku Cancer Center, Matsuyama 791-0280, Japan; ^8^Department of Gastroenterology and Hepatology, Kurashiki Central Hospital, Kurashiki 710-8602, Japan; ^9^Department of Internal Medicine, Okayama Saiseikai General Hospital, Okayama 700-8511, Japan; ^10^Department of Pathology, Okayama University Graduate School of Medicine, Dentistry and Pharmaceutical Sciences, Okayama 700-8558, Japan

## Abstract

**Background:**

Clinical characteristics and prognosis of patients with a solitary Peutz-Jeghers polyp (PJP) have not been fully investigated.

**Methods:**

Solitary PJP was diagnosed when a single hamartomatous lesion was identified in the gastrointestinal tract of patients without mucocutaneous pigmentation or a family history of Peutz-Jeghers syndrome. We retrospectively reviewed 51 patients (32 men and 19 women) with a solitary PJP and analyzed the sex, age at diagnosis, endoscopic features, and outcomes in this patient group. The STK11/LKB1 germline mutation was not investigated in any of the patients.

**Results:**

The mean age of the 51 patients was 66.1 years. The polyp was found in the duodenum (*N* = 10), jejunum (*N* = 2), cecum (*N* = 2), transverse colon (*N* = 5), sigmoid colon (*N* = 21), or rectum (*N* = 11). Most of the polyps presented as a pedunculated lesion (*N* = 40), followed by semipedunculated (*N* = 9) and sessile (*N* = 2) morphologies. The mean size of a solitary PJP was 15.6 mm (range: 5 to 33 mm). During a mean endoscopic follow-up period of 4.5 years (range: 0.1 to 16.1 years), no recurrence was identified. Eighteen of the enrolled patients had a history of cancer or concomitant cancer. Five patients died due to non-gastrointestinal-related causes. No additional cancer or death directly related to solitary PJP was observed.

**Conclusions:**

Solitary PJPs did not recur in this study. Although examination of the entire gastrointestinal tract using esophagogastroduodenoscopy, enteroscopy, and colonoscopy is desirable to exclude Peutz-Jeghers syndrome, follow-up endoscopy after endoscopic polyp resection may be unnecessary, once the diagnosis of a solitary PJP is made.

## 1. Introduction

Peutz-Jeghers syndrome is an autosomal dominant genetic disorder characterized by the development of multiple polyps in the gastrointestinal tract in association with patches of hyperpigmentation in the mouth and on the hands and feet [1, 2]. The gastrointestinal polyps found in patients with Peutz-Jeghers syndrome are hamartomatous and are typically characterized by distinctive arborization of smooth muscle within the lamina propria [3]. These hamartomatous lesions can present as solitary polyps in the gastrointestinal tract in patients without mucocutaneous pigmentation and are termed solitary Peutz-Jeghers polyps (PJPs).

Owing to infrequency, the clinical characteristics and outcome of cases with a solitary PJP have not been fully investigated. In this report, we identified 51 patients with a solitary PJP from 9 institutions and summarized the endoscopic findings, presence or absence of malignant tumors in the follow-up period, and prognoses.

## 2. Methods

**Figure 1:**
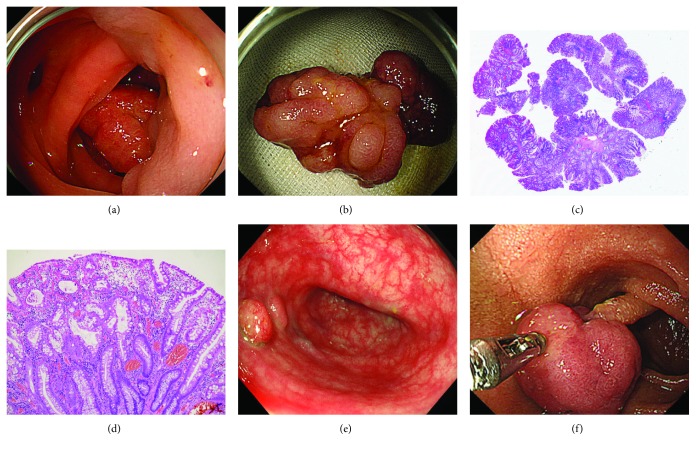
A solitary PJP in the sigmoid colon was endoscopically resected with a snare device (a, b). Pathological analysis revealed that the resected polyp was hamartomatous, showing arborization of smooth muscle within the lamina propria (c, d). A solitary PJP showing sessile morphology was observed in the sigmoid colon (e). In another patient, a pedunculated polyp in the duodenum was diagnosed as a solitary PJP (f).

A solitary PJP was defined as a single hamartomatous lesion detected in the gastrointestinal tract of patients without a family history of Peutz-Jeghers syndrome or mucocutaneous pigmentation. Representative pathological features of PJP are extensive smooth-muscle proliferation and an elongated arborized pattern of polyp formation (Figure 1). Peutz-Jeghers syndrome was diagnosed when the patient had any of the following clinical criteria, and these patients were excluded from the study: 2 or more histologically confirmed PJPs, any number of PJPs detected in a person whose family history included 1 close relative with Peutz-Jeghers syndrome, characteristic mucocutaneous pigmentation in a person whose family history included 1 close relative with Peutz-Jeghers syndrome, and any number of PJPs in a person who also had characteristic mucocutaneous pigmentation [4–6].

We retrospectively reviewed 51 patients (32 men and 19 women) with a solitary PJP and analyzed the sex, age at diagnosis, endoscopic features, and outcomes in this patient group. Letters of inquiry regarding patients with solitary PJP were sent from the Department of Gastroenterology and Hepatology, Okayama University Graduate School of Medicine, Dentistry and Pharmaceutical Sciences, to 8 collaborating institutions. Patients with a pathologically diagnosed PJP in the gastrointestinal tract were included. Finally, a total of 51 patients diagnosed with a solitary PJP between January 1999 and April 2018 were identified and were retrospectively registered in this study. Three of the 51 patients examined also participated in our previous study [7]. Although the STK11/LKB1 germline mutation has been reported to be specific for Peutz-Jeghers syndrome, the presence or absence of the mutation was not investigated in any of the patients.

The follow-up period was defined as the time from diagnosis of PJP to death from any cause or the last hospital visit. The endoscopic follow-up period was defined as the time from resection of a solitary PJP to the last endoscopic examination to evaluate the intestinal segment with the PJP. Statistical analyses were performed using JMP 14.0.0 software (SAS Institute, Inc., Cary, NC, USA), and 1-way analysis of variance followed by a Tukey-Kramer post hoc test for multiple comparisons. *P* < 0.05 was considered to indicate a statistically significant difference. The present study was approved by the Ethics Committee of Okayama University Hospital and adhered to the Declaration of Helsinki.

## 3. Results

Characteristics of the enrolled patients and the location, type, and size of the polyps are shown in Table 1. The solitary PJP was found in the duodenum, jejunum, cecum, transverse colon, sigmoid colon, or rectum, while no cases of polyps in the esophagus, stomach, ileum, ascending colon, and descending colon were observed. Most of the polyps presented as a pedunculated lesion, followed by semipedunculated and sessile morphologies.  Table 2 shows the distribution in the gastrointestinal tract and PJP morphology. With respect to polyp size in each intestinal segment, one-way analysis of variance followed by Tukey-Kramer post hoc testing found no differences in polyp sizes across locations.

Among those who underwent endoscopic examination, 48 patients (94%) had colonoscopy and 33 (65%) had esophagogastroduodenoscopy. Enteroscopy was performed in only 4 patients (8%): 3 had video capsule enteroscopy alone and 1 patient with a PJP in the jejunum underwent peroral double-balloon enteroscopy and video capsule enteroscopy. In the latter patient, the jejunal polyp was diagnosed during video capsule enteroscopy and endoscopically resected during a double-balloon enteroscopy. The other patient with a PJP in the jejunum underwent colectomy for ascending colon cancer, and the polyp was diagnosed during a laparotomy via digital examination of the small intestine. Segmental resection of the jejunum was performed to remove the polyp. Intraoperative endoscopy revealed no other polyps in the small intestine.

Twenty-one malignancies were diagnosed in 18 patients (35%) before the solitary PJP was identified. Sixteen of the malignancies were cancers also seen in patients with Peutz-Jeghers syndrome: 12 cancers of the gastrointestinal tract, including gastric cancer (*N* = 5), cecal cancer (*N* = 1), colon cancer (*N* = 4), and rectal cancer (*N* = 2). Breast cancer was found in two patients whereas lung and thyroid cancers were seen in one case each. In addition, prostate cancer (*N* = 3), hypopharyngeal cancer (*N* = 1), and liver cancer (*N* = 1) were found in the enrolled patients. These had been diagnosed prior to the identification of PJP in all patients, and no cancers were diagnosed during the follow-up after resection of the solitary PJP.

The solitary PJP was endoscopically resected and endoscopically followed up in 25 patients (49%). Follow-up endoscopy was not performed in the other 26 patients. During a mean endoscopic follow-up of 4.5 years (range: 0.1 to 16.1 years), no recurrence was identified. Patients were followed up for an average of 3.8 years (range: 0.0 to 18.8 years) to determine prognosis. Five patients died as follows: hypopharyngeal cancer (*N* = 1), interstitial pneumonia (*N* = 1), pulmonary thrombosis (*N* = 1), sepsis (*N* = 1), or unknown cause (*N* = 1). All other patients were alive as of the last scheduled follow-up.

## 4. Discussion

To our knowledge, this is the largest study to date on patients with a solitary PJP. In the 51 patients in this study, the sigmoid colon was most frequently involved, followed by the rectum, duodenum, transverse colon, jejunum, and cecum. Overall, a solitary PJP was found in the large intestine in more than three-quarters of the patients, e.g., cecum, transverse colon, sigmoid colon, and rectum (*N* = 39, 77%). Although gastric and ileal involvement was not observed in this study, cases with a solitary PJP in the stomach [8] and ileum [9] have been reported. However, to our knowledge, no patients have had a solitary PJP in the esophagus. Patients with Peutz-Jeghers syndrome typically have multiple hamartomatous polyps in the stomach, small intestine, and/or large intestine [2]. Thus, as in Peutz-Jeghers syndrome, the esophagus does not seem to be affected by solitary PJPs.

Although 5 patients died of causes unrelated to PJP, the remaining 46 were alive as of the last scheduled follow-up. In addition, no recurrence was detected on follow-up endoscopy in 25 patients. Oncel et al. reviewed 8 patients with a solitary PJP and found no recurrences during a median of 11.5 years (range: 3 to 22 years) [10]. They also noted that 3 patients died of causes other than PJP, while the other 5 were alive. These results suggest that a solitary PJP does not recur. When a single polyp showing pathological features consistent with PJP is identified in the gastrointestinal tract, physical examination to check for mucocutaneous pigmentation and a review of family history are essential. In addition, we considered that examination of the entire gastrointestinal tract using esophagogastroduodenoscopy, enteroscopy, and colonoscopy is desirable to exclude Peutz-Jeghers syndrome. Once the diagnosis of a solitary PJP is made, follow-up endoscopy after endoscopic resection of the polyp may be unnecessary.

Patients with Peutz-Jeghers syndrome reportedly have a lifetime cumulative risk of up to 93% for development of malignancies, such as colorectal, breast, small bowel, gastric, and pancreatic cancers [2, 11, 12]. Ishida et al. reviewed 583 Japanese patients with Peutz-Jeghers syndrome reported in the literature and estimated the cumulative risk of a malignant tumor as 83.0% at 70 years of age [6]. In the present study, the polyps were often detected in patients with cancer: patients had gastrointestinal, prostate, breast, lung, thyroid, hypopharyngeal, and liver cancers. However, these had been diagnosed prior to identification of a PJP in all enrolled patients. Although no additional cancer or mortality directly related to solitary PJPs was observed in this study, the follow-up time was rather short in some cases and only half of the patients underwent a follow-up endoscopy. Therefore, further investigation is required to determine the relationship between solitary PJPs and malignancies.

This study had several limitations. First, not all patients had endoscopic evaluation of the entire gastrointestinal tract. In particular, the small intestine was not evaluated in 47 patients, although polyposis in the small bowel is frequently observed in Peutz-Jeghers syndrome. Second, the presence of STK11/LKB1 germline mutations, which are specific for Peutz-Jeghers syndrome, was not investigated in solitary PJP patients. In Japan, unfortunately, genetic testing is virtually unavailable in clinical settings, because it is not covered by health insurance. As a result, several patients may have had undiagnosed multiple PJPs or STK11/LKB1 germline mutations; thus, true Peutz-Jeghers syndrome may have been misdiagnosed as solitary PJP. Third, malignant diseases had been diagnosed in a subset of the enrolled patients prior to the detection of PJP. Fourth, follow-up endoscopy was performed in 25 patients, while endoscopy examinations were not repeated in the remaining 26 patients. In addition, the observation period of 3.8 years is relatively short. A complete follow-up with a longer observation period is desirable to accurately estimate the recurrence rate, prognosis, and the frequency of malignant tumors.

## 5. Conclusions

We retrospectively investigated 51 patients with a solitary PJP. Although 5 patients died of causes unrelated to a solitary PJP, the other patients were alive as of the last scheduled follow-up. During a mean endoscopic follow-up of 4.5 years, no recurrence was observed, indicating that solitary PJPs do not recur.

## Figures and Tables

**Table 1:** Clinical characteristics of study subjects.

	*N*
Sex	
Male	32 (63%)
Female	19 (37%)
Mean age (years)	66.1 (range: 32-92)
Location	
Esophagus	0
Stomach	0
Duodenum	10 (20%)
Jejunum	2 (4%)
Ileum	0
Cecum	2 (4%)
Ascending colon	0
Transverse colon	5 (10%)
Descending colon	0
Sigmoid colon	21 (41%)
Rectum	11 (22%)
Mean size ± SD (mm)	15.6 ± 7.1 (range: 3-33)
Morphology	
Pedunculated	40 (78%)
Semipedunculated	9 (18%)
Sessile	2 (4%)
Evaluation of recurrence	
Done	25 (49%)
Not done	26 (51%)
Recurrence of PJ polyps	0
Mean endoscopic follow-up period (years)	3.1 (range: 0.1-16.1)
Outcome	
Alive	46 (90%)
Died of other cause^∗^	5 (10%)
Mean follow-up period (years)	3.8 (range: 0.0-18.8)

^∗^Hypopharyngeal cancer, interstitial pneumonia, pulmonary thrombosis, sepsis, and unknown cause.

**Table 2:** Gastrointestinal tract involvement and PJP morphology.

	Duodenum	Jejunum	Cecum	Transverse colon	Sigmoid colon	Rectum	Total
Ip	7	2	1	5	18	7	40
Isp	3	0	0	0	2	4	9
Is	0	0	1	0	1	0	2
Total	10	2	2	5	21	11	51

Ip: pedunculated; Isp: semipedunculated; Is: sessile.

## Data Availability

The clinical data used to support the findings of this study are available from the corresponding author upon request.
